# Spatio-temporal learning from molecular dynamics simulations for protein–ligand binding affinity prediction

**DOI:** 10.1093/bioinformatics/btaf429

**Published:** 2025-08-19

**Authors:** Pierre-Yves Libouban, Camille Parisel, Maxime Song, Samia Aci-Sèche, Jose C Gómez-Tamayo, Gary Tresadern, Pascal Bonnet

**Affiliations:** Institute of Organic and Analytical Chemistry (ICOA), UMR7311, Université d’Orléans, CNRS, Pôle de chimie rue de Chartres, 45067 Orléans Cedex 2, France; Institute for Development and Resources in Intensive Scientific Computing (IDRIS), CNRS, Rue John Von Neumann, 91403 Orsay Cedex, France; Institute for Development and Resources in Intensive Scientific Computing (IDRIS), CNRS, Rue John Von Neumann, 91403 Orsay Cedex, France; Institute of Organic and Analytical Chemistry (ICOA), UMR7311, Université d’Orléans, CNRS, Pôle de chimie rue de Chartres, 45067 Orléans Cedex 2, France; Computational Chemistry, Janssen Research & Development, Janssen Pharmaceutica N. V, B-2340 Beerse, Belgium; Computational Chemistry, Janssen Research & Development, Janssen Pharmaceutica N. V, B-2340 Beerse, Belgium; Institute of Organic and Analytical Chemistry (ICOA), UMR7311, Université d’Orléans, CNRS, Pôle de chimie rue de Chartres, 45067 Orléans Cedex 2, France

## Abstract

**Motivation:**

The field of protein–ligand binding affinity prediction continues to face significant challenges. While deep learning (DL) models can leverage 3D structural information of protein–ligand complexes, they perform well only on heavily biased test sets containing information leaked from training sets. This lack of generalization arises from the limited availability of training data and the models’ inability to effectively learn from protein–ligand interactions. Since these interactions are inherently time-dependent, molecular dynamics (MD) simulations offer a potential solution by incorporating conformational sampling and providing interaction rich information.

**Results:**

We have developed MDbind, a dataset comprising 63 000 simulations of protein–ligand interactions, along with novel neural networks capable of learning from these simulations to predict binding affinity. By utilizing MD as data augmentation, our models achieved state-of-the-art performance on the PDBbind v.2016 core set and an external test set, the free energy perturbation (FEP) dataset. Additionally, when trained on the full MD simulations, the models demonstrated less biased predictions.

**Availability and implementation:**

The code for neural networks is available at https://github.com/ICOA-SBC/MD_DL_BA. The models, the results and the training/validation/test sets are available for download at https://zenodo.org/records/10390550. The MDbind trajectories are being transferred to the MDDB: https://mdposit.mddbr.eu/#/browse?search=MDBind .

## Graphical Abstract

**Graphical Abstract:**
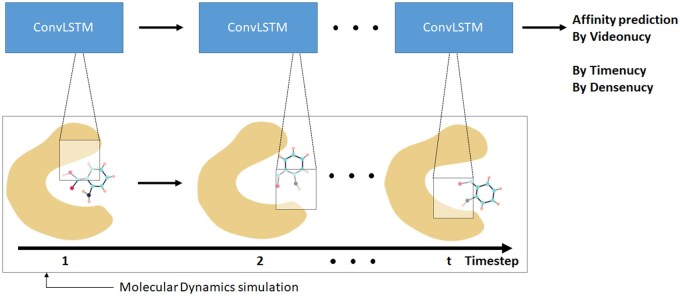

## 1 Introduction

The accurate prediction of the binding affinity of protein–ligand complexes is a major goal in drug design. Such predictions serve as key criteria used to guide the selection of molecules for bioactivity measurement or to recommend new molecules to be synthetized by medicinal chemists. To prioritize molecules by their potential activity, a usual approach in the molecular modeling field is to dock them in the binding site of a protein; the resulting docking poses are then evaluated by their fit and the associated scoring function. Various scoring functions have been implemented for this purpose (Li *et al.* 2019); however, these scoring functions rarely provide a correlation with binding affinity. In the last decade, machine learning scoring functions were able to outperform other methods when benchmarked on datasets composed of experimentally determined protein–ligand complexes with known binding affinities (Su *et al.* 2019). Within AI methods, classical methods, such as random forest combined with extended connectivity interaction features, have established state-of-the-art performance. On the other hand, the rise of deep learning (DL) allowed creating architectures that take advantage of the 3D information and atom connectivity to carry out predictions. These models were mainly developed by using convolutional neural network (CNN) (Jiménez *et al.* 2018, Stepniewska-Dziubinska *et al.* 2018, Wu *et al.* 2018, Li *et al.* 2019, Francoeur *et al.* 2020, Hassan-Harrirou *et al.* 2020, Kwon *et al.* 2020, Liu *et al.* 2021) or graph neural network (GNN) (Feinberg *et al.* 2018, Lim *et al.* 2019, Son and Kim 2021, Moon *et al.* 2022, Volkov *et al.* 2022). The advantage of learning from 3D structures directly is to prevent models from using expertly crafted descriptors that carry human biases, as well as providing the framework to introduce inductive biases by having extensive control of the training process. A review of their performance is provided by Meli *et al.* (2022).

Unfortunately, published performances are generally overly optimistic as models were evaluated on data for which biases have been identified (Volkov *et al.* 2022). Additionally, when applied on new or more challenging test sets, the models are not predictive, hence limiting their use in real-world scenarios like hit optimization. Poor generalizability can be attributed to several factors. Lack and quality of the data stand out as the major limitation. Most binding affinity prediction models are trained on experimental data that are compiled in the PDBbind dataset (Wang *et al.* 2004). In its version 2020 (v.2020), it is composed of 19 443 complexes with known binding affinities an increase of 6135 complexes compared to v.2016. Unfortunately, this quantity of data is considerably lower than in other fields where DL is applied successfully, such as image classification, where typical datasets comprise on the order of millions of instances, such as ImageNet (Deng *et al.* 2009), currently composed of 14 000 000 images. The limitation on data availability, especially for challenging problems like binding affinity prediction, leads to low model performance and overfitting issues. Furthermore, the PDBbind dataset is sparse compared to drug discovery lead optimization use cases, lacking series of ligands targeting the same protein and series of proteins interacting with the same ligand. Thus, the protein–ligand affinity training is oblivious to the details that drive structure-activity relationships and existence of activity cliffs (Volkov *et al.* 2022). These limitations result in statistical models that typically do not learn from the actual protein–ligand interactions but instead memorize biases in the data, including patterns of affinity that correlate solely with the target proteins or ligands (Volkov *et al.* 2022, Libouban *et al.* 2023). Some models have been shown to perform similarly to conventional ligand-based quantitative structure-activity relationship (QSAR) (Sieg *et al.* 2019, Scantlebury *et al.* 2020); therefore, alternative methods need to be investigated to obtain models able to learn on the protein–ligand interactions.

On the other hand, classical physics-based methods calculate the absolute binding free energies (ABFE) for the association of protein and ligand (ΔG), like LIE (Hansson *et al.* 1998), MMPB(GB)SA (Hou *et al.* 2011), and variants of free energy perturbation (FEP) (Khalak *et al.* 2021, Gapsys *et al.* 2022, Chen *et al.* 2023). These calculations are performed via molecular dynamics (MD) simulations that assess different conformational states of the protein and the ligand. ABFE FEP is approaching the accuracy of relative binding free energy (RBFE) calculations [ΔΔG around 1 kcal/mol root mean square error (RMSE)]. Although, the evaluation of the free energies requires heavy computation, especially with the FEP variants. Therefore, it is more suitable to apply these tools for a lead optimization process rather than for virtual screening.

Data augmentation has proven to be an effective strategy to mitigate some of the limitations of DL models in the field (Pereira *et al.* 2016, Ragoza *et al.* 2017, Xie *et al.* 2020, Moon *et al.* 2022). Since it is not possible to rapidly increase the amount of experimental data due to time and cost, many researchers have turned to MD simulations as a viable alternative. Indeed, MD simulations provide temporal information about protein–ligand interactions, which is crucial for accurately predicting protein–ligand binding affinity, given the inherently dynamic nature of these interactions. Consequently, two MD simulation datasets, called PLAS-5k (Korlepara *et al.* 2022) and MISATO (Siebenmorgen *et al.* 2024), were introduced to address this need. PLAS-5k (Korlepara *et al.* 2022) is composed of five simulation replicates lasting 4 ns each for a total of 5000 complexes, with 2000 of them having known binding affinities. OnionNet (Zheng *et al.* 2019) was trained on this dataset and achieved a high correlation coefficient $R_{\text{CV}}^{2}$ of 0.947 with a 10-fold cross-validation, although with a poor RMSE of 5.7 kcal/mol (approx. 4 log units of error if converted to pK affinities). On the other hand, MISATO (Siebenmorgen *et al.* 2024) encompasses MD simulations of 8 ns performed on 16 972 complexes from the PDBbind (v.2019).

Multiple machine learning models were implemented to train on 4D descriptors extracted from MD simulations (Jamal *et al.* 2019, Gu *et al.* 2023). These 4D descriptors, such as MD fingerprints (Riniker 2017), can be generated by calculating 3D molecular descriptors [e.g. solvent accessible surface area (SASA), radius of gyration, or potential energies] for each frame of the simulation and subsequently calculating their average or concatenating them into a vector. In addition, a few DL models were also developed, including ProtMD (Wu *et al.* 2022), an E(3)-equivariant graph matching network that underwent pre-training on 62 800 frames obtained from MD simulations lasting 100 ns on 64 protein–ligand complexes. After fine-tuning using 90% of the ATOM3D (Townshend *et al.* 2020) binding affinity dataset, this model achieved a correlation coefficient of 0.6 and a RMSE of 1.4 pK_i_ on the 10% remaining dataset. In another study from Berishvili *et al.* (2019), a MLP (multi-layer perceptron), a CNN and a LSTM (long short-term memory) models were trained on MD simulations of 2 ns performed on 356 complexes from the PDBbind dataset. Using molecular descriptors extracted from the MD simulations, they achieved correlation coefficients of 0.70 and 0.68 with the CNN and the LSTM, respectively, on the CSAR (community structure-activity resource) test set. The Dynaformer (Min *et al.* 2024) method is a graph transformer model that was pre-trained on frames extracted from MD simulations, which lasted 10 ns and were performed on 3218 complexes from the PDBbind. It was subsequently fine-tuned on PDBbind complexes and reached a correlation coefficient of 0.86 and a RMSE of 1.1 pKi on the PDBbind v.2016 core set.

In a similar way, we have applied a protocol combining MD simulations with DL algorithms. For this purpose, a dataset of MD simulations called MDbind was generated. This dataset encompasses 63 000 simulations achieved by conducting 10 replicate simulations of 10 ns per complex, for 6300 complexes. Beyond simply expanding the dataset, the MD simulations provide physical insights into protein–ligand interactions behavior over time. They are based on an all-atom parameterized system and capture the dynamics of the protein–ligand complex. One of the objectives of this protocol was to enable DL models to discern differences between high- and low-affinity ligands and to capture the variations of their interactions, where most of the models trained on static single 3D protein–ligand complexes often fail to capture. Subsequently, we developed a series of neural network architectures to harness this data for the task of binding affinity prediction. These neural networks architectures are designed to take advantage of MD simulation data in two distinct ways. First, frames can be extracted from simulations and considered independently from each other as new structures while being labeled with the binding affinity of the initial complex. Hence, MD simulations act as a data augmentation method compatible with the neural network architecture. Second, it is possible to train models from the whole simulations. In this case, the simulations are labeled with the binding affinity of the initial complex. We coined the term “spatio-temporal learning” for this training methodology. This method exploits the temporal information contained sequentially in the frames of the simulations. For that purpose, we have developed two neural networks, a long-term recurrent convolutional network (LRCN) and a convolutional long short-term memory (ConvLSTM), both able to carry out binding affinity predictions by analyzing simulations. Models trained with MD data augmentation outperformed current state-of-the-art methods, while spatio-temporal learning approaches are reaching performance similar to state-of-the-art methods.

## 2 Materials and methods

### 2.1 MD data structure

The MDbind dataset is composed of 63 000 MD simulations obtained by carrying out 10 simulations of 10 ns on 6300 complexes from the PDBbind (Wang *et al.* 2004). Simulation replicas were performed for each complex to enhance the sampling of conformational space and reduce uncertainty in binding affinity predictions (Adler and Beroza 2013, Wright *et al.* 2014, 2019, Wan *et al.* 2020). We chose to use 10 replicates based on the recommendations of Adler and Beroza (2013), which prioritize sampling effectiveness while maintaining a reasonable computational cost.

Simulations were performed on complexes with fully resolved proteins, prioritizing those in the PDBBind refined set, a high-quality subset of PDBBind, followed by complexes from the rest of the dataset. The distributions of protein families within the PDBbind (v.2020), the MDbind, and the PDBbind v.2016 core set are provided in Supplementary Information (Supplementary Fig. S1, available as supplementary data at *Bioinformatics* online). This information provides a clearer assessment of the diversity of protein families and the inherent biases in these datasets. Peptides accounted for 20% of all ligands in MDbind, which is consistent with the proportions observed in PDBbind. Proteins and ligands were prepared (protonated at neutral pH, partial charge added using AM1-BCC method…) with AmberTools20 (Pearlman *et al.* 1995) including Antechamber (Wang *et al.* 2006), leap and parmchk2, as well as PDB4Amber. The ff14SB (Maier *et al.* 2015) and the general amber force field (gaff) (Wang *et al.* 2004) were applied to proteins and ligands, respectively. Explicit solvent was used using TIP3P (Mark and Nilsson 2001) and counter-ions (Na^+^, Cl^−^) added. Complexes were minimized, heated and equilibrated for 2 ns. Simulations were performed using Amber20 (Pearlman *et al.* 1995), in NPT with the SHAKE algorithm (Ryckaert *et al.* 1977) and using particle mesh Ewald MD (PMEMD) engine (Essmann *et al.* 1995). A new seed was generated for each replicate by performing an additional short equilibration step.

Considering simulation speed and storage size, frames were recorded every 200 picoseconds, resulting in 50 frames per simulation. This approach produced a total of 3 000 000 frames from 63 000 simulations. We did not perform any sub-selection of the frames provided to the neural networks because we aimed to use all available data for training the models. To improve efficiency, only the pockets are fed to the neural networks. Following our previous work (Libouban *et al.* 2023), the pockets were defined by selecting the residues at 12 Å from the geometric center of the ligand in the crystallographic poses. Pocket extraction and frame processing were performed using Pymol script (DeLano 2002), Pytraj (Roe and Cheatham 2013, Nguyen and Roe 2016) and MDAnalysis (Gowers *et al.* 2016). The dataset used as input for the neural networks contains the 3D coordinates of atoms from both pockets and ligands. The dataset was compressed in both HDF5 and NumPy format. The datasets were split with a ratio of 80/20 between training and validation set. All extracted frames or simulations of a complex are exclusively allocated to either the training or the validation set.

For the MD data augmentation, the sets used in this work are composed of:

- validation set: the crystallographic poses of 1198 complexes randomly selected from the refined set (4852 complexes) and 585 372 frames extracted from 11 940 simulations (1194 complexes)
- training set: the crystallographic poses of 16 076 complexes selected from the general set (17 679 complexes) without the validation set (1 198 complexes) and 2 340 237 frames extracted from 47 501 simulations (4751 complexes). We did not conduct subsampling; therefore, the models were trained on all available frames.
- test set: the crystallographic poses of 285 complexes of the PDBbind v.2016 core set

For the spatio-temporal learning, the sets are composed of:

- training set: 46 632 simulations performed on 4753 complexes
- validation set: 11 668 simulations performed on 1179 complexes
- test set: 830 simulations performed on 83 complexes of the PDBbind v.2016 core set. This test set is referred to as MDbind test set. The 41 500 frames extracted from this test set are also used to evaluate MD data augmentation methods.

The FEP dataset (Wang *et al.* 2015) has been used as an additional external test set. It comprises 200 protein ligand complexes from 8 different proteins (BACE, CDK2, JNK1, MCL1, PTP1B, Thrombin, TYK2, and P38) and a limited amount of ligand scaffolds. Each series of molecules targets the same protein, while exhibiting a wide range of binding affinities. This provides the opportunity to test the models in a different scenario closer to a real-world problem, where biases identified in the core set are less present. Even if there are no redundancies in complexes between the FEP dataset and the PDBbind, it is important to note that 10 ligands from the FEP dataset (5%) were found in PDBbind. Furthermore, all proteins from the FEP dataset were found in PDBbind.

### 2.2 Neural networks

Pafnucy (Stepniewska-Dziubinska *et al.* 2018), a well-known 3D CNN developed for binding affinity predictions, was used as a reference to develop the neural networks presented in this paper. Convolutions are performed over a box that is created around the ligand accounting for the pocket information. By default, each side of the cubic box measures 25 Å. The box space is discretized in voxels of 1 Å³. Voxels in contact with atoms were assigned atomic features. Each atomic feature corresponds to a channel in the CNN, and the convolutions are performed on the voxels. Nineteen features were used to describe atoms, such as atom types, partial charge, atom hybridization. The detail is provided in Pafnucy paper (Stepniewska-Dziubinska *et al.* 2018). As CNNs are sensitive to rotational and translational motion in space, it is usual to train models on the 24 rotations of the cube (90° rotations). To save computational power, we decided to train the models on only one random rotation of each complex, as it provided similar performance from our tests.

**Figure 1.**
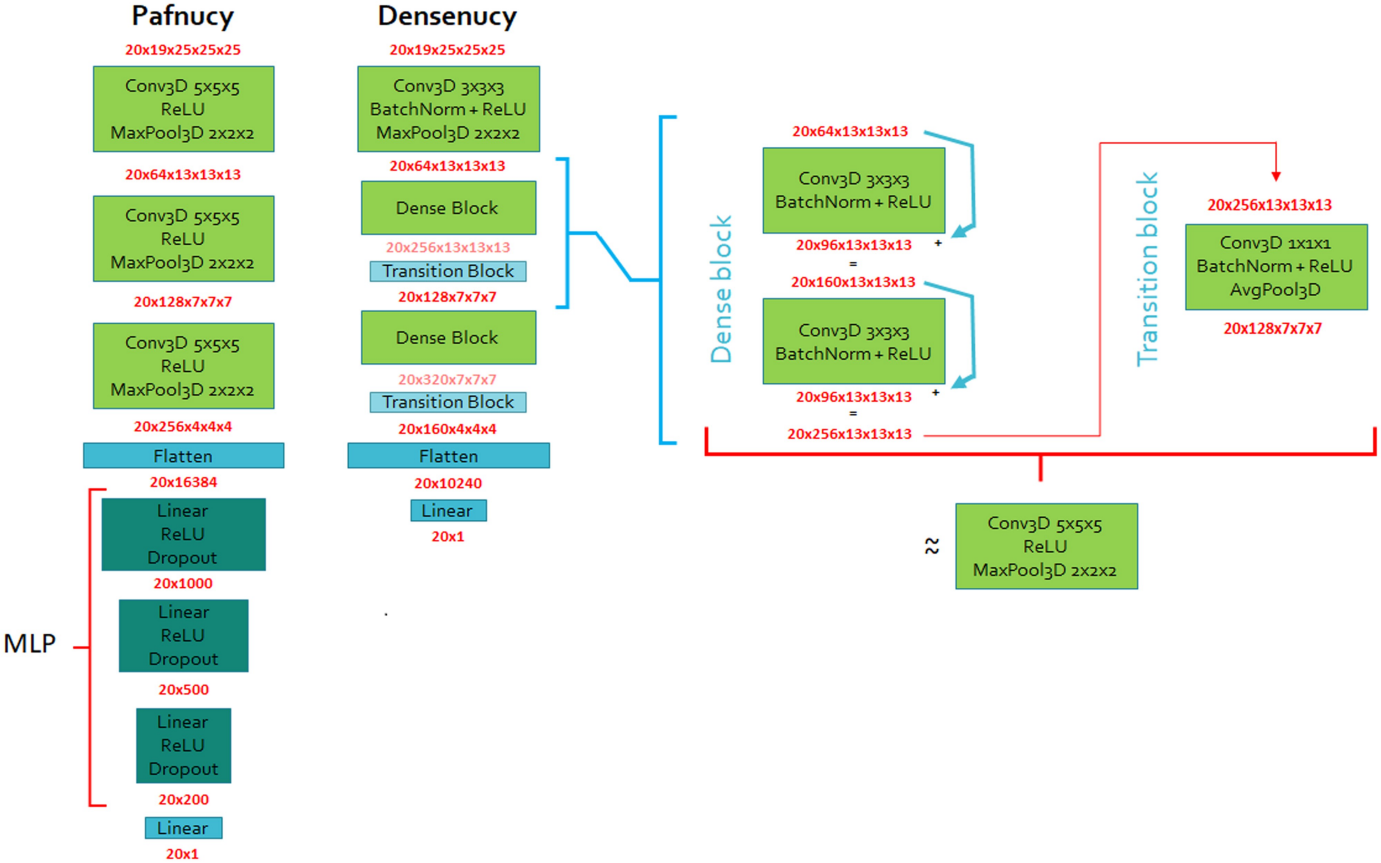
Comparison of the architecture of Pafnucy and Densenucy. A detailed breakdown of the dense and transition blocks is displayed. The dimensions of layers’ input and output are displayed in red. The first number represents the batch size, followed by the number of channels and the dimension of the box. The flatten layer compresses the spatial dimensions into the channel dimension.

First, we ported Pafnucy from Tensorflow 1.2 to PyTorch, and following the example set by Imrie *et al.* (2018), we have developed an updated version called Densenucy, by replacing convolutional blocks by dense blocks (Fig. 1). The dense block is a modern framework that allows reaching better performance in computer vision. It facilitates scaling up the neural networks size by adding convolutional layers. Furthermore, it mitigates the loss of information during the convolution. This is achieved by adding the input of layers to the output, therefore preserving the initial information. Additionally, the size of filters was reduced to lower memory load and computational cost. As a result, Densenucy is more compact than Pafnucy with a lower number of parameters. Thereafter, a long-term recurrent convolutional network (LRCN) and a convolutional long short-term memory (ConvLSTM) were developed to train from the whole simulations, transitioning from using 3D to 4D input data. Both process the information of each atom, including their features and positions, for each frame of the simulation.

**Figure 2.**
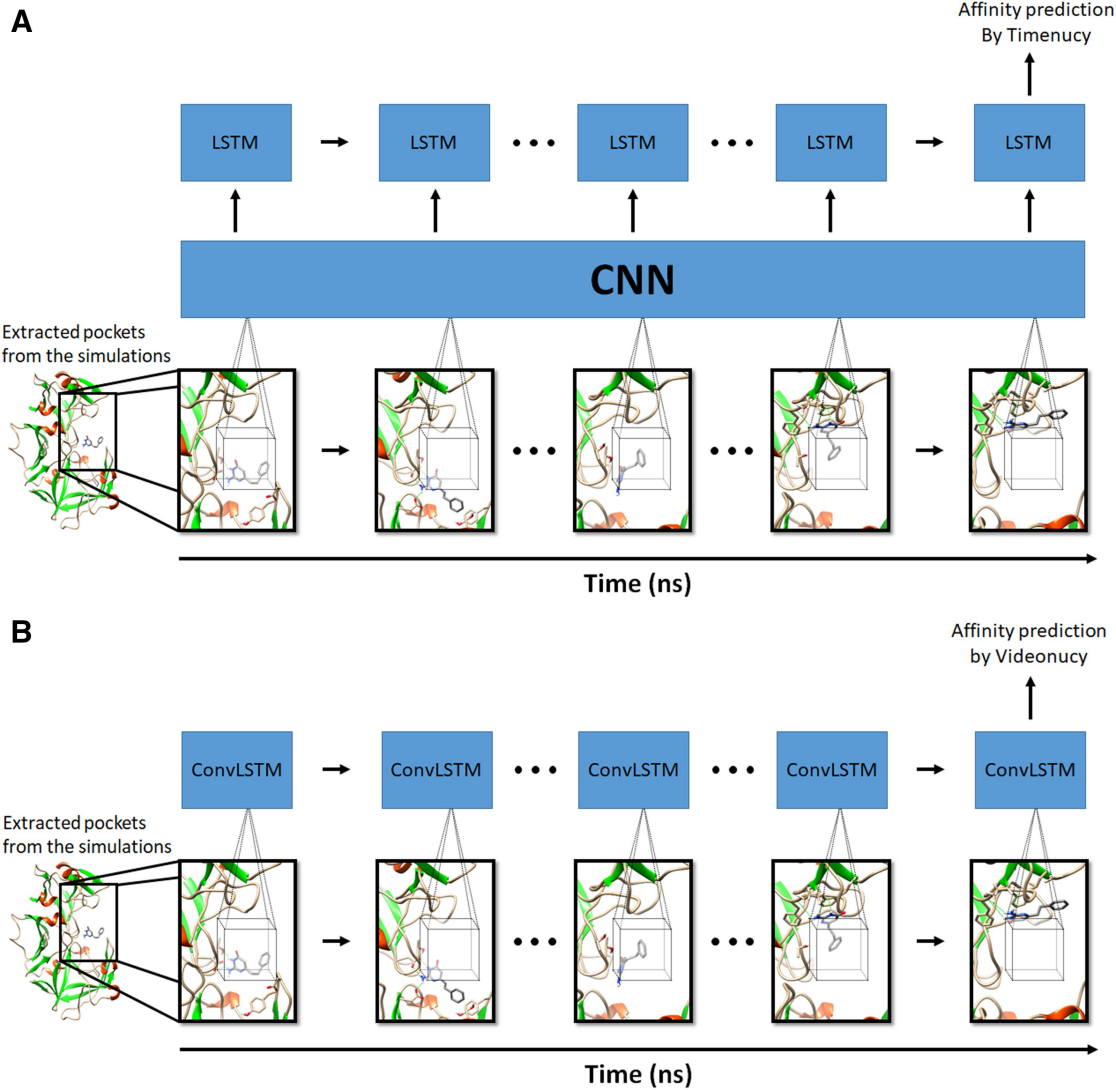
Workflows of Timenucy (A) and Videonucy (B). Timenucy is a long-term recurrent convolutional network (LRCN). Videonucy is a convolutional long short-term memory (ConvLSTM). In both workflows, all frames from a simulation are analyzed in order to carry a prediction of the binding affinity.

The LRCN architecture was introduced for video activity recognition (Donahue *et al.* 2017). It is composed of a CNN followed by a LSTM. LRCN analyzes videos frame by frame, examining each image individually, before carrying a final prediction. In a similar fashion, we created Timenucy, a LRCN capable of processing 4D data by using MD simulations as input. It works in two steps: first, the CNN performs convolutions on all the frames, and then the concatenated output is sent to the LSTM for the final analysis (Fig. 2A).

**Figure 3.**
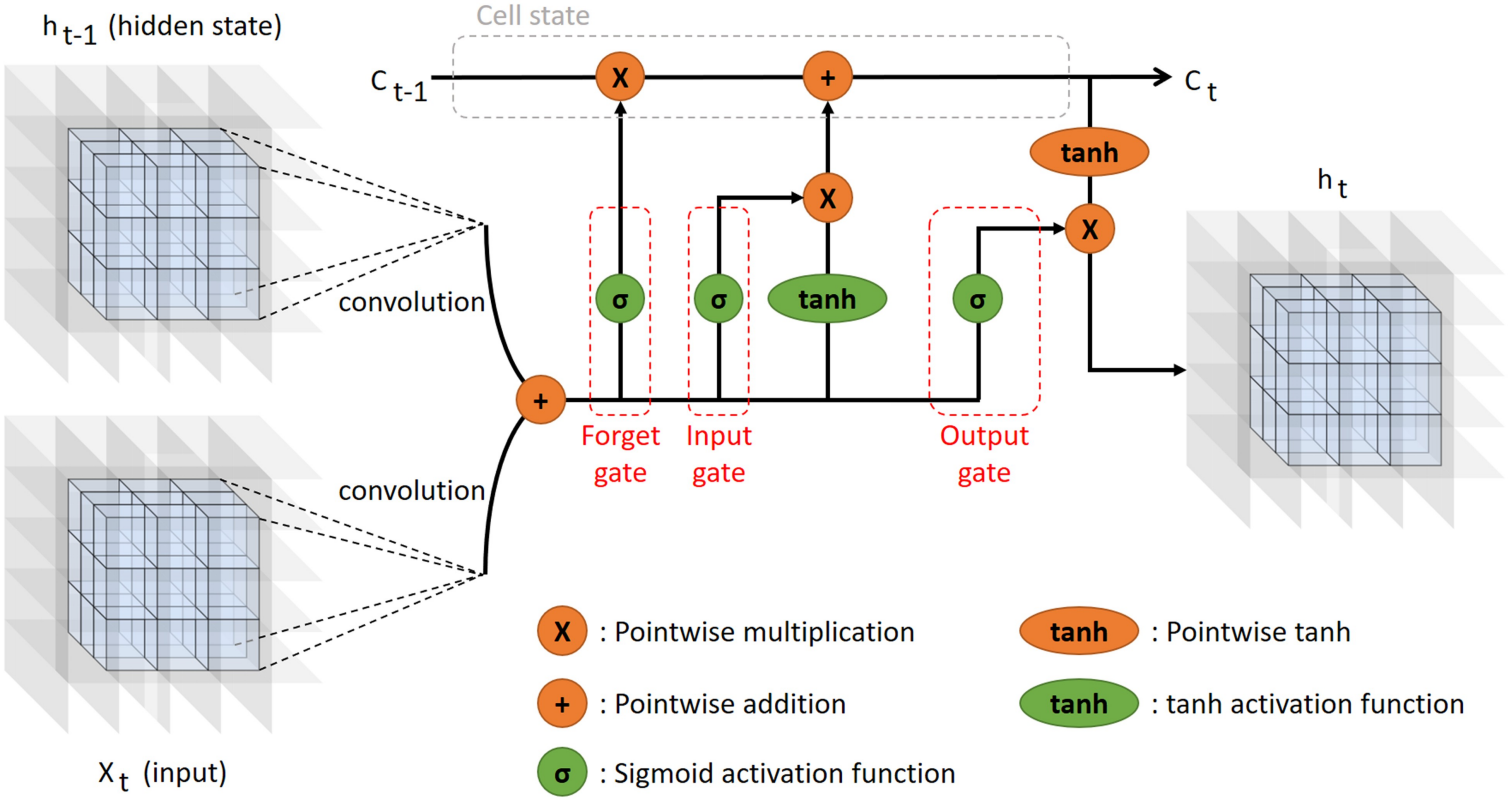
The intrinsic mechanism of the ConvLSTM applied on 4D data.

The convolutional LSTM, called Videonucy (Fig. 2B), combines the convolution process with the LSTM mechanism (Fig. 3) (Yuan *et al.* 2018). The advantage of such an approach is that it tracks the movement of each atom across the simulation, thus fully using the 4D data that is provided to it. The original implementation of the convolutional LSTM comes from Shi *et al.* (2015). We adapted the code from https://github.com/ndrplz/ConvLSTM_pytorch.

Details of the implementations of Timenucy and Videonucy can be found in the Supplementary data (Supplementary Fig. S2, available as supplementary data at *Bioinformatics* online). Additional information regarding the hyperparameters of all models can be found in the Supplementary Materials.

The performance of the models was measured using Pearson’s correlation coefficient (R) and root mean square error (RMSE) for regression, and Spearman’s correlation coefficient (ρ) for the classification task.

## 3 Results

First, we compared the performance of models trained with random versus systematic rotations of PDBbind complexes on the PDBbind v.2016 core set. For Pafnucy and Densenucy, no improvement in performance was observed with rotational data augmentation (Supplementary Fig. S3, available as supplementary data at *Bioinformatics* online). Consequently, we opted to perform random rotations in further training to reduce the computational load. In Supplementary Fig. S4, available as supplementary data at *Bioinformatics* online, we also included an analysis of Pafnucy’s performance in a “Leave One Target Out” setting by removing kinase family from the training set, demonstrating the limitations of the current models in generalization.

### 3.1 MD data augmentation

**Figure 4.**
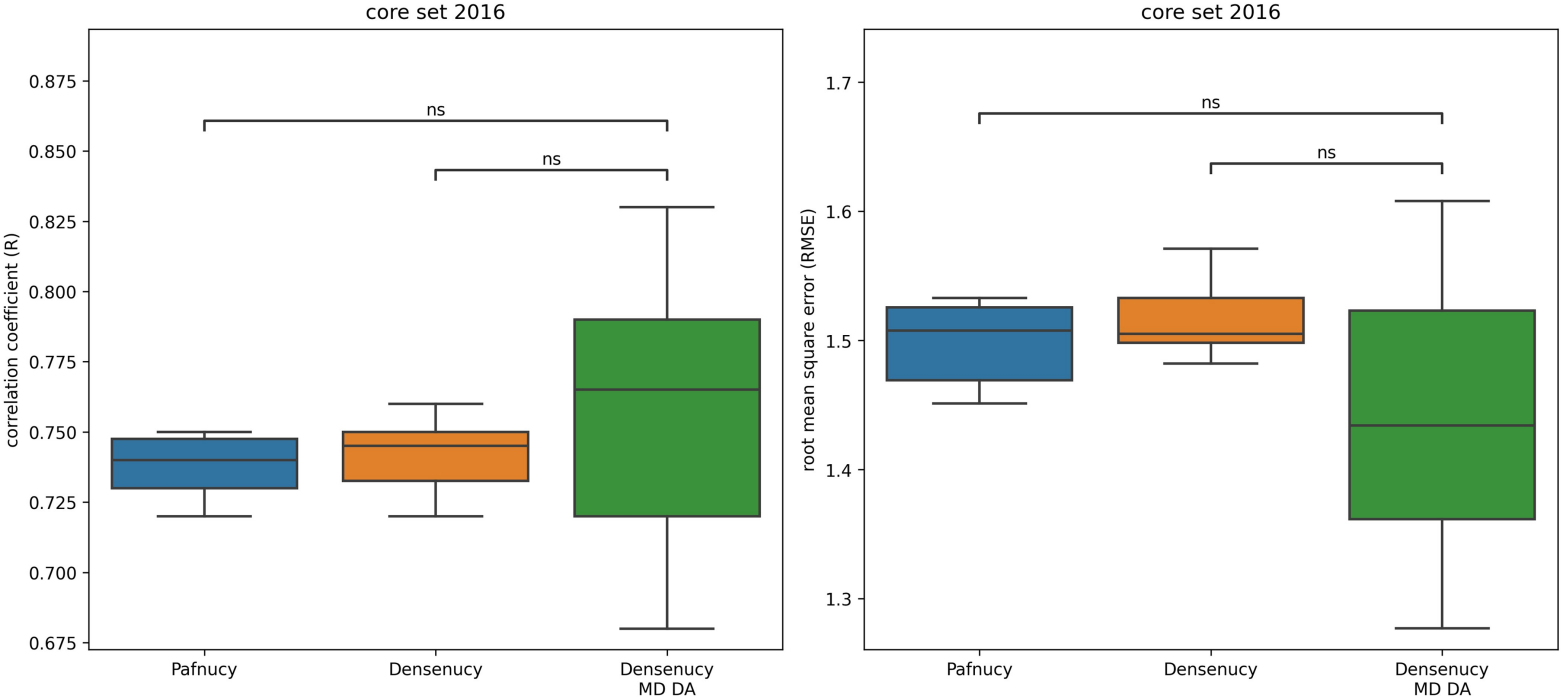
Comparison of the performance of Densenucy with or without data augmentation. Pafnucy performance without data augmentation on the same pockets was added for benchmark purpose. Results are evaluated on the PDBbind v.2016 core set. For each training setting, the boxplot displays the results over 10 replicate models. “MD DA” refers to molecular dynamics data augmentation. The following *P*-values correspond to the annotations on the plots: ns: 5.00 × 10^−2^ < *P* ≤ 1.00 × 100.

We assessed the impact of MD data augmentation on model performance by training models either exclusively on the crystallographic poses from PDBbind or with additional frames extracted from simulations (Fig. 4). With MD data augmentation, Densenucy was trained on a dataset of 3 000 000 structures instead of just 18 000. Performance was evaluated on the PDBbind v.2016 core set. Incorporating MD data augmentation during the training of Densenucy resulted in the highest performance, with an R-value of 0.83 and an RMSE of 1.28. In contrast, Pafnucy without data augmentation attained an R-value of 0.75 and an RMSE of 1.45, consistent with those published in the original paper. (Stepniewska-Dziubinska *et al.* 2018). These findings highlight the potential benefits of MD data augmentation for enhancing the performance of certain models, bringing their performance in line with state-of-the-art methods (Meli *et al.* 2022).

### 3.2 Spatio-temporal learning

While MD data augmentation has shown promise in enhancing model performance, this approach only partially helps models capture the dynamics of protein–ligand complexes. This limitation stems from treating the extracted frames as independent of one another. To fully leverage the temporal information embedded in MD data, we developed spatio-temporal learning models: a long-term recurrent convolutional network (LRCN) named Timenucy and a convolutional LSTM (ConvLSTM) named Videonucy.

**Figure 5.**
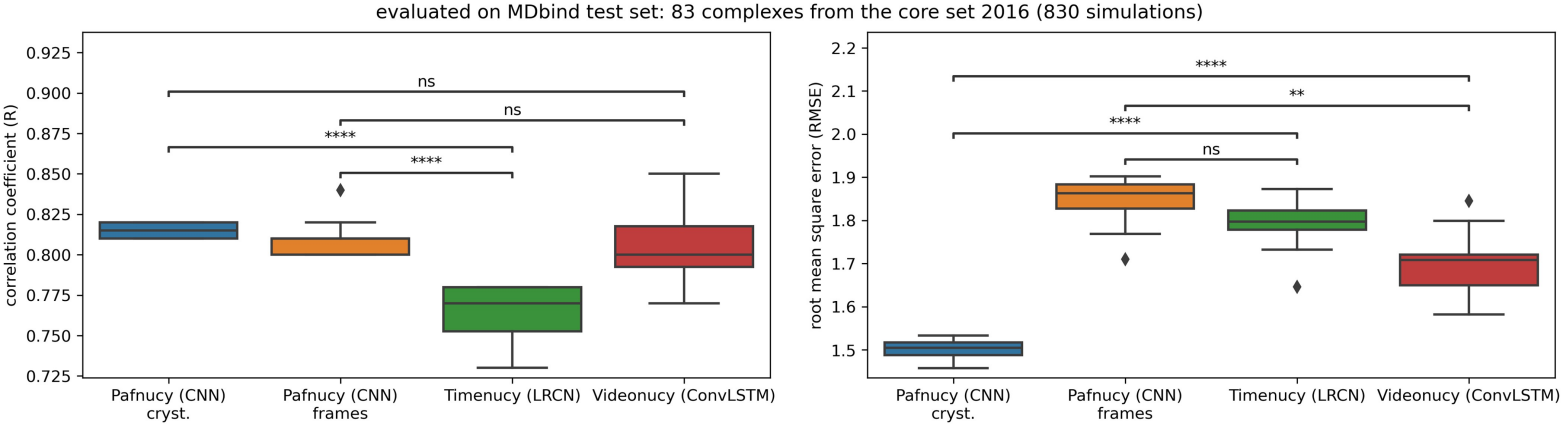
Comparison of the performance of Pafnucy (CNN) and the spatio-temporal models Timenucy (LRCN) and Videonucy (ConvLSTM). The models were trained on 4753 complexes, validated on 1179 complexes and evaluated on the MDbind test set composed of 83 complexes from the PDBbind v.2016 core set. Pafnucy was trained on crystallographic poses [Pafnucy (CNN) Cryst.] or on frames [Pafnucy (CNN) frames] extracted from the simulations. Timenucy and Videonucy were evaluated on 830 simulations. For each training setting, the boxplot displays the results over 10 replicate models. The following *P*-values correspond to the annotations on the plots: ****: *P* ≤ 1.00 × 10^−4^, **: 1.00 × 10^−3^ < *P* ≤ 1.00 × 10^−2^, ns: 5.00 × 10^−2^ < *P* ≤ 1.00 × 100, and black squares (◆) are possible outliers.

The performance of these models was evaluated against Pafnucy on the MDbind test set (Fig. 5). The performance was also measured on the crystal structure of the 83 complexes from the PDBbind v.2016 core set. Both Pafnucy and the spatio-temporal models were trained, validated, and tested on the same complexes. To generate a single prediction for each complex, predictions were averaged across the 10 simulations for Timenucy and Videonucy, or across the 500 frames for Pafnucy. Although Timenucy and Videonucy showed lower or similar performance compared to Pafnucy, the RMSE of Pafnucy significantly increased when evaluated on frames instead of crystallographic poses. Under these conditions, Videonucy achieved a lower RMSE than Pafnucy.

### 3.3 Evaluation of biases

Recent studies (Moon *et al.* 2022, Volkov *et al.* 2022) have highlighted that many deep learning models trained on the PDBbind dataset are biased, suggesting that these models may be relying on inherent biases in the data rather than learning from protein–ligand interactions to predict binding affinity. To address these concerns, we implemented a method to assess the models’ ability to learn from these interactions. Model performance was evaluated by training on datasets where either the protein or the ligand information was removed. Then, we calculated the gap in prediction accuracy between learning on the full complex versus only one of the molecular partners. A small gap would indicate that the model is not effectively using the information from the entire complex.

**Figure 6.**
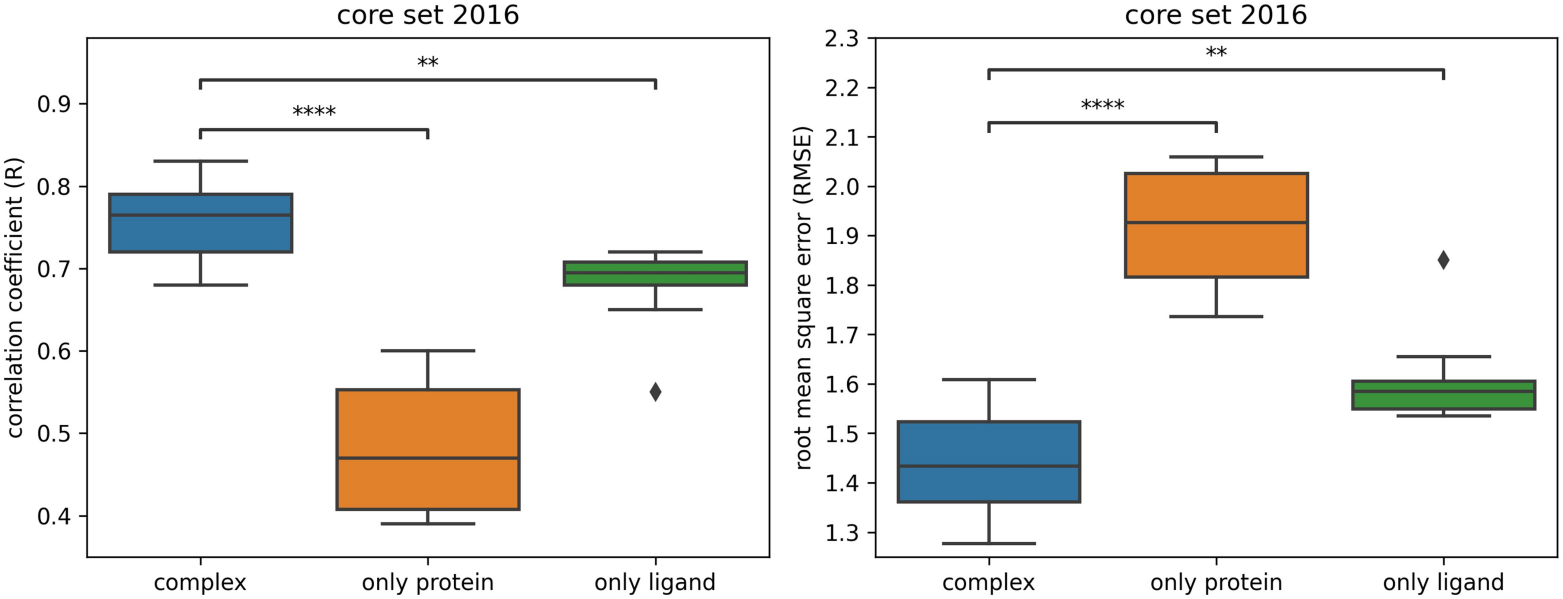
Comparison of the performance of Densenucy using MD data augmentation (MD DA) when trained without proteins or ligands. Performance was assessed on the PDBbind v.2016 core set, while removing the protein (“only ligand”) or the ligand (“only protein”) information during the training of the models. The following *P*-values correspond to the annotations on the plots: ****: *P* ≤ 1.00 × 10^−4^, **: 1.00 × 10^−3^ < *P* ≤ 1.00 × 10^−2^, and ◆ are possible outliers.

Densenucy shows better performance when trained on both the pockets and ligands together, rather than on just one of the partners (Fig. 6). The difference in performance between training on the full complex versus solely on proteins or ligands was assessed by comparing the mean performance across 10 model replicates for each training condition. This resulted in a performance gap of ΔR_prot_ = 0.27 when Densenucy was trained on proteins only. The gap in performance when training Densenucy with ligands only was of ΔR_lig_ = 0.08. For comparison, the performance gaps for Pafnucy without MD data augmentation were ΔR_prot_ = 0.11 and ΔR_lig_ = 0.13 (Libouban *et al.* 2023).

**Figure 7.**
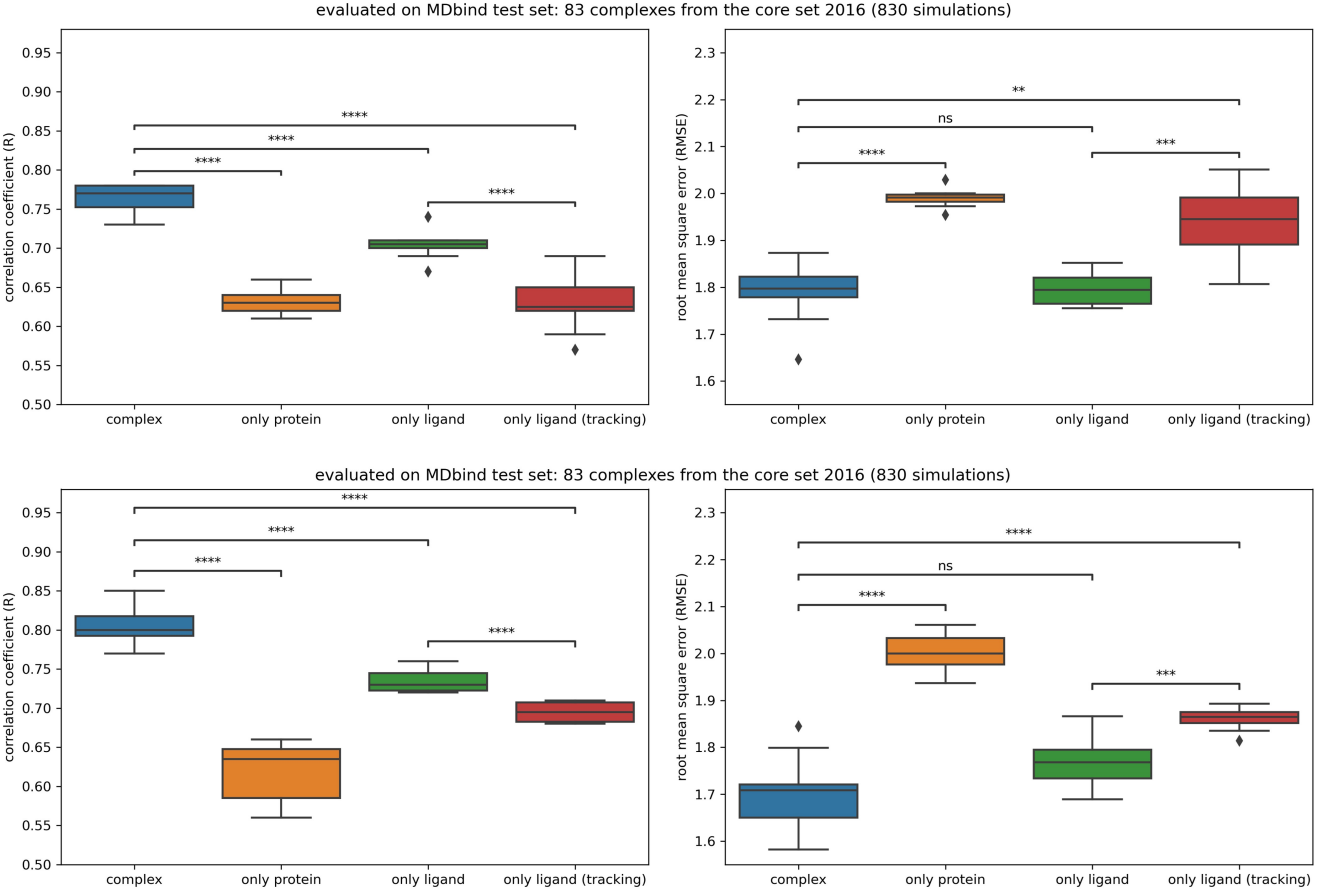
Comparison of the performance of spatio-temporal learning methods, Timenucy (top) and Videonucy (bottom) when trained without protein or ligand information. Performance was assessed on the MDbind test set, composed of 830 simulations from 83 complexes from the PDBbind v.2016 core set. The protein or the ligand were removed from the frames according to the training method. Models with “complex”, “only protein” and “only ligand” are trained with boxes centered on pockets. The “only ligand (tracking)” models use boxes centered on the ligands. The following *P*-values correspond to the annotations on the plots: ****: *P* ≤ 1.00 × 10^−4^, ***: 1.00 × 10^−4^ < *P* ≤ 1.00 × 10^−3^, **: 1.00 × 10^−3^ < *P* ≤ 1.00 × 10^−2^, ns: 5.00 × 10^−2^ < *P* ≤ 1.00 × 100, and ◆ are possible outliers.

Timenucy and Videonucy exhibit significantly better performance when trained on complexes compared to training on ligands or proteins alone (Fig. 7). The gaps in performance in using the whole complex and the protein only are ΔR_prot_ = 0.13 and ΔR_prot_ = 0.19 for Timenucy and Videonucy, respectively. Similarly, the gaps in performance between using the whole complex and the ligand only are ΔR_lig_ = 0.06 and ΔR_lig_ = 0.07 for Timenucy and Videonucy, respectively. Despite the significant difference, models trained solely on ligands exhibited performance levels close to those trained on entire complexes. One explanation for this could be that the models learn mostly bias from training set using the ligand structure.

To verify this hypothesis we used new boxes, named “only ligand (tracking)”, by centering them on the ligand for each frame of the simulation. Previously, we were using boxes centered on a fixed pocket composed of predefined amino acids located around the ligand of the initial pose. Thus, in “only ligand (tracking)”, the ligands are always located in the center of the boxes while initially ligands could be found on the extremities or even out of the boxes. Using the boxes centered on the ligands (tracking mode), we observe that the performance of spatio-temporal models trained without proteins are significantly lower than with boxes centered on the pockets (ΔRlig_tracking = 0.13 and ΔRlig_tracking = 0.11 for Timenucy and Videonucy, respectively).

One interpretation could be that valuable information is derived from the ligand stability within the binding site, which can improve binding affinity predictions. This suggests that the spatio-temporal models leverage insights from the movements of the ligands throughout the boxes, thereby gaining an understanding of ligand dynamic behavior. This claim is supported by the plot of complexes’ affinity as a function of ligand movement in the binding pocket (maximum RMSD per ligand) (Supplementary Fig. S5, available as supplementary data at *Bioinformatics* online), which shows that only 36% of ligands in low-affinity complexes are deemed stable (with a maximum ligand root mean square deviation (RMSD) less than 2 Å), while it increases to 58% for high-affinity complexes. Therefore, a valuable signal related to ligand stability could enhance binding affinity predictions through the analysis of MD simulations.

### 3.4 Extra metrics and external test set

To better compare the performance of the different neural networks, we have measured their ability to classify complexes in function of their affinity by using the Spearman’s coefficient. We present Spearman’s coefficients calculated on the entire test sets as well as those calculated for each cluster of the test set, which are then averaged.

The performance (R, RMSE, and ρ) of each model is summarized in the Table 1. In addition to Figs 4 and 5, which illustrate the distribution of metrics across the 10 models, this table employs a consensus method that averages predictions from the 10 model replicates to present the best performances for all models.

**Table 1.** Performance of models on the PDBbind v.2016 core set and the MDbind test set.^a^

Models	R	RMSE	ρ all	ρ cluster
Pafnucy	0.76	1.45	0.75	0.59
Pafnucy MD DA	0.70	1.62	0.68	0.56
Densenucy	0.77	1.48	0.76	0.62
Densenucy MD DA	**0.81**	**1.36**	**0.81**	**0.67**

Pafnucy reduced	0.83	**1.47**	0.82	0.74
Timenucy	0.78	1.78	**0.83**	0.66
Videonucy	**0.84**	1.66	**0.83**	**0.76**

a MD DA stands for MD data augmentation. Pafnucy and Densenucy were evaluated on the PDBbind v.2016 core. Pafnucy reduced, Timenucy and Videonucy were evaluated on the MDbind test set. The best results are displayed in bold.

As the performance of models trained on the PDBbind and evaluated on the PDBbind v.2016 core set are biased, we decided to further benchmark our models using an external test set, the FEP dataset, (Wang *et al.* 2015) as displayed in Table 2. This dataset comprises similar ligands with different binding affinities for the same proteins. It allows assessing the models’ ability to predict activity cliffs. Several machine learning scoring functions were evaluated on the FEP dataset, including RF-score (Ballester and Mitchell 2010), a random forest-based model, and K_DEEP_ (Jiménez *et al.* 2018), a state-of-the-art CNN model.

**Table 2.** Performance of models on the FEP dataset.^a^

	RF-score		K_DEEP_		Pafnucy		Pafnucy MD DA		Densenucy MD DA	
	R	RMSE	R	RMSE	R	RMSE	R	RMSE	R	RMSE
Bace	−0.12	**0.65**	−0.06	0.84	**0.33**	0.75	−0.38	0.74	−0.05	0.72
CDK2	−0.24	1.05	0.69	1.26	0.21	0.85	**0.70**	0.78	0.62	**0.69**
Jnk1	0.61	**0.50**	**0.69**	1.18	0.34	0.66	0.68	0.55	−0.66	0.88
MCL1	0.51	0.99	0.34	1.04	0.46	1.00	0.29	0.89	**0.59**	**0.71**
PTP1B	0.26	0.90	0.58	0.93	**0.81**	0.97	0.66	**0.84**	0.76	0.85
Thrombin	0.08	0.71	0.58	0.44	**0.67**	**0.39**	**0.67**	0.75	0.50	0.95
Tyk2	0.41	0.94	−0.22	1.13	−0.55	1.24	0.22	**0.91**	**0.69**	1.18
p38	0.48	0.90	0.36	1.57	0.63	0.95	0.47	0.87	**0.66**	**0.62**
average	0.25	0.83	0.37	1.05	0.36	0.85	**0.41**	**0.79**	0.39	0.83
Weighted average	0.28	0.84	0.33	1.08	**0.40**	0.88	0.33	0.80	0.38	**0.78**
Median	0.34	0.9	0.47	1.09	0.40	0.90	0.56	0.81	**0.61**	**0.79**
Whole dataset	NA	NA	NA	NA	0.45	0.90	0.57	0.81	**0.59**	**0.79**

a The results of RF-score and K_DEEP_ were published by Jiménez *et al.* (2018) “MD DA” stands for MD data augmentation. The best results are displayed in bold.

Pafnucy and Densenucy trained with MD data augmentation (MD DA) outperform the other models on most targets. We note that Densenucy was able to drastically enhance predictions for specific protein clusters, such as p38, where the performance improved from R = 0.36 and RMSE = 1.57 with KDEEP to R = 0.66 and RMSE = 0.62 with Densenucy MD data augmentation. Given that we achieved robust performance on a dataset comprising similar ligands targeting the same protein with varying affinities, it opens up the possibility of using such tools in lead optimization.

In Supplementary Fig. S6, available as supplementary data at *Bioinformatics* online, we further assess the ΔRlig of Densenucy MDDA on the FEP dataset and demonstrate that this gap is increased compared to the PDBbind v.2016 core set. This suggests that the models trained with MD augmentation generalize better due to their reduced dependency to ligand memorization, effective in a biased test set like PDBbind v.2016 core set, but not in a real-world scenario posed by the FEP dataset.

## 4 Conclusion

Despite advancements in neural network architectures, models trained and evaluated on commonly used datasets are hitting a performance ceiling in predicting the binding affinity of protein–ligand complexes. The limitations of traditional test sets—primarily due to dataset biases and data leakage—have led to overly optimistic performance evaluations (Buttenschoen *et al.* 2023, Durairaj *et al.* 2024, Gómez-Tamayo *et al.* 2024, Li *et al.* 2024). When assessed on more challenging and less biased test sets, these models often fail to generalize effectively. This highlights the inherent difficulty in training models that can truly grasp the physics of protein–ligand interactions and make accurate binding affinity predictions. To address these challenges, significant efforts have been directed toward augmenting training datasets. Initially, data augmentation methods were developed mainly based on molecular docking protocols (Li *et al.* 2016, Francoeur *et al.* 2020, Son and Kim 2021, Boyles *et al.* 2022). More recently, data augmentation was performed using molecular dynamics simulations (Korlepara *et al.* 2022, Min *et al.* 2024, Siebenmorgen *et al.* 2024).

In a similar fashion, to provide dynamic information on protein–ligand complexes, we set up a new MD data augmentation protocol. Beyond simply increasing data volume, this approach aims to provide models with new insights to overcome previously mentioned limitations. First, we created MDbind, a dataset comprising 63 000 simulations obtained by performing 10 simulations of 10 ns on 6300 PDBbind complexes. Then, we evaluated the performance of 3D-CNN named Pafnucy and Densenucy, on the PDBbind core set (Su *et al.* 2019) and the FEP dataset (Wang *et al.* 2015).

Although Densenucy with data augmentation outperformed Pafnucy in both regression and ranking tasks on the PDBbind core set, MD data augmentation did not improve Pafnucy’s performance on the PDBbind v.2016 core set, where it maintained its original levels. However, the MD data augmentation enhanced Pafnucy’s performance on the more challenging FEP dataset. We speculate that MD data augmentation fails to enhance performance on the PDBbind v.2016 core set because the dataset’s biases already enable high baseline performance, which does not always correlate to good generalizability. However, on more demanding external test sets like the FEP dataset, MD data augmentation helps produce models that generalize better and achieve superior results.

Furthermore, we developed two neural networks that perform spatio-temporal learning on the whole MD simulations; a LRCN called Timenucy and a ConvLSTM referred to as Videonucy. Due to computation time constraints, models were trained in a non-optimal way using randomly selected simulation of each complex per epoch. The idea was to design a proof of concept that it is possible to learn from all the information contained in MD simulation, by keeping the temporal link between frames. Models were trained on MDbind and they achieved modest performance on the MDbind test set, which is composed of simulations performed on complexes from the PDBbind core set. Although, it seems that these models are able to learn from ligand behavior in the pocket, as they get better performance by using boxes centered on the pockets rather than on the ligands when solely using the ligand. These methods could be useful in challenging scenarios, such as activity cliffs, where current models struggle because they are mainly acting as QSAR. Recognizing the PDBbind core set’s limited relevance to real-world scenarios, we plan to evaluate the generalizability of spatio-temporal learning methods using the FEP dataset. Future work will focus on expanding the MDbind dataset, minimizing bias in the training data, and enhancing the models to improve their ability to generalize.

Overall, we developed two methodologies to train DL models from MD simulations, namely the MD data augmentation and spatio-temporal learning, which can be applied in different stages of the drug design process. On the one hand, the MD data augmentation approach aims at improving the performance of neural networks initially developed to analyze static data. Models trained with this method can be easily applied in a virtual screening process to score docking poses. On the other hand, the spatio-temporal learning method can only be applied on complexes that have undergone MD simulations beforehand. If proven able to generalize, such methods would be preferably applied in a hit-to-lead or lead optimization phase, to select promising molecules among reduced number of ligands.

## Supplementary Material

btaf429_Supplementary_Data

## Data Availability

The data underlying this article are available in Zenodo, at https://zenodo.org/records/10390550. The code is available in GitHub at https://github.com/ICOA-SBC/MD_DL_BA. The MDBind trajectories are available at https://mdposit.mddbr.eu/#/browse?search=MDBind.
