# Supplementary material for: Spatio-temporal learning from molecular dynamics simulations for protein–ligand binding affinity prediction
Source: Bioinformatics. 2025 Aug 19;41(8):btaf429. doi: 10.1093/bioinformatics/btaf429 (PMC12371333; doi:10.1093/bioinformatics/btaf429)
Supplement: btaf429_Supplementary_Data [file btaf429_supplementary_data.zip › Supplementary_Materials_MDBind.docx]

**Supplementary Information: Spatio-temporal learning from molecular dynamics simulations for protein-ligand binding affinity prediction**

Pierre-Yves Libouban, Camille Parisel, Maxime Song, Samia Aci-Sèche, Jose C. Gómez-Tamayo, Gary Tresadern, and Pascal Bonnet


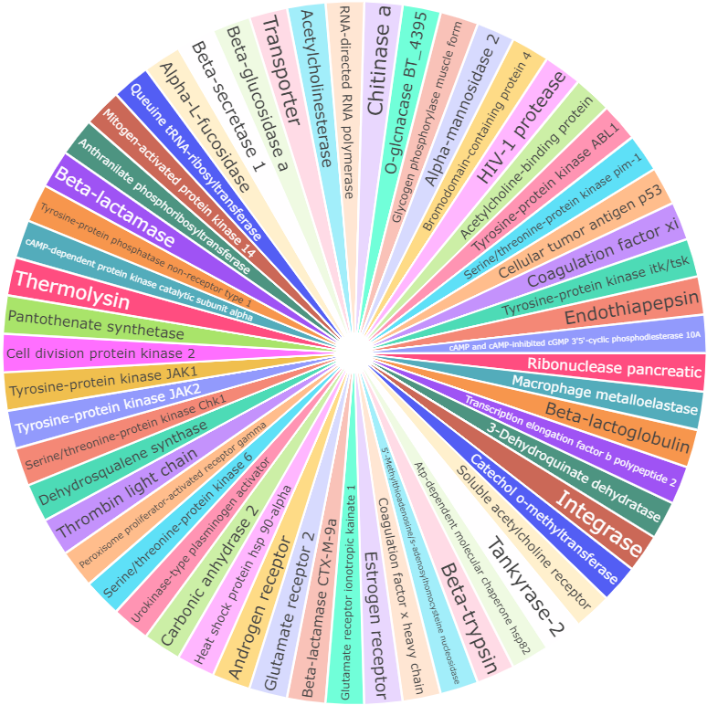

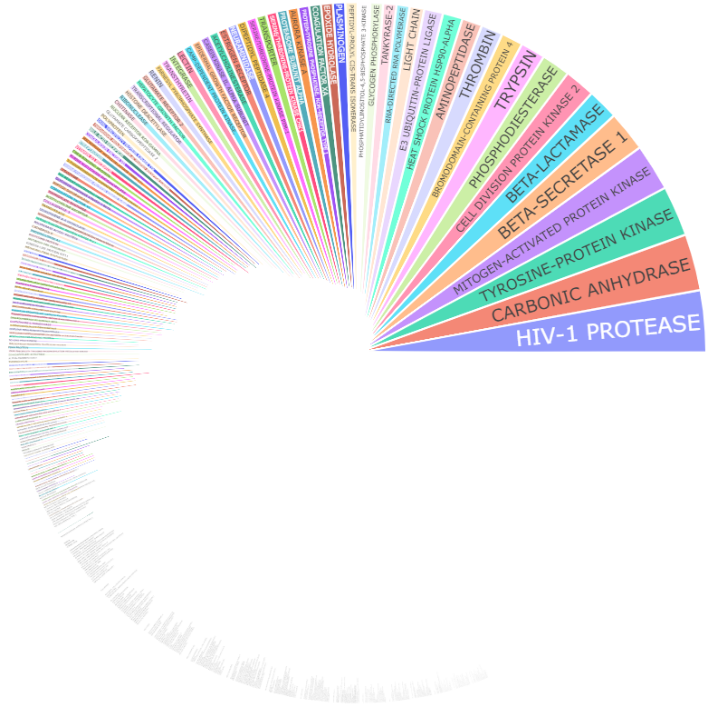


PDBbind v.2016 core set set

PDBbind (v.2020)

MDbind


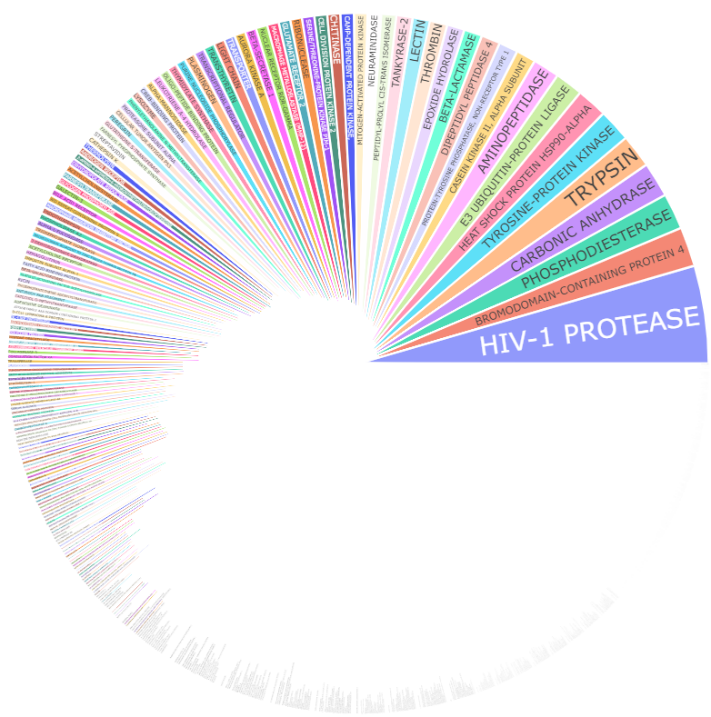


**Fig. S1: Piecharts of the distribution of protein families across PDBbind (v.2020), PDBbind v.2016 core set, and MDbind.** Complexes were assigned to protein families based on the CATH classification system (Orengo *et al.* 1997). The size of each protein family representation is proportional to the number of complexes in the datasets.


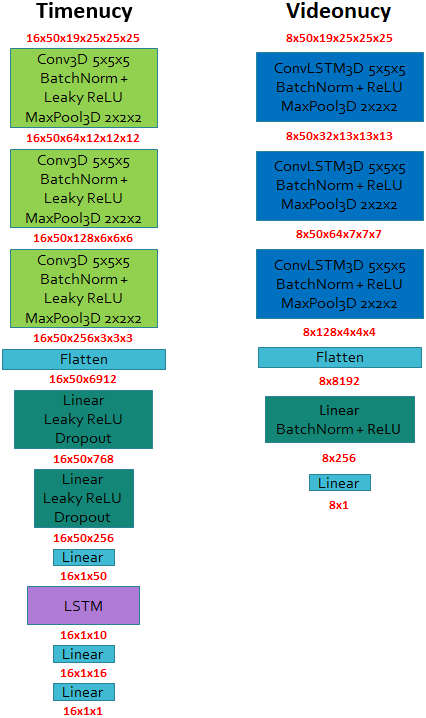


**Fig. S2. Detailed architecture of Timenucy (LRCN) and Videonucy (ConvLSTM).** The convolutional layers are shown in light green, while the ConvLSTM layers are in dark blue. The LSTM is in purple, and the multi-layer perceptron (MLP) is in dark green. The flatten and output layers (also output of the CNN and LSTM) are in cyan. The dimensions of layers’ input and output are displayed in red. The first number represents the batch size, followed by the number of frames, the number of channels and the dimension of the box. The flatten layer compresses the spatial dimensions into the channel dimension.

Hyperparameters:

The dropout was implemented for regulation purposes, allowing models to generalize better. It was set to 0.5 for Proli’s MLP (three FC layers). For Timenucy, a dropout of 0.5 was applied on the layers of the FCN, while it was set to 0.2 for the LSTM layer.

The Adam optimizer was used on all neural networks with a weight decay of 10-4. The optimizer was applied with a learning rate of 10-4 for Timenucy, while the others used a learning rate of 10-5. A learning rate scheduler was also added to Timenucy with 10 epochs of warmup. It was implemented to help models escape from local minima during training.

Aside from Timenucy, the rectified linear unit (ReLU) activation function was used everywhere. In the case of Timenucy, leaky ReLU were used with the CNN and the FCN. The leaky ReLU is a modified version of the ReLU, which helps mitigate the vanishing gradient problem. A batch size of 20 was used with Pafnucy and Densenucy, while it was set to 16 and 8 for Timenucy and Videonucy respectively.

The models were trained for a maximum of 200 epochs, with training halted if performance on the validation set did not improve for 20 consecutive epochs to speed up the process.


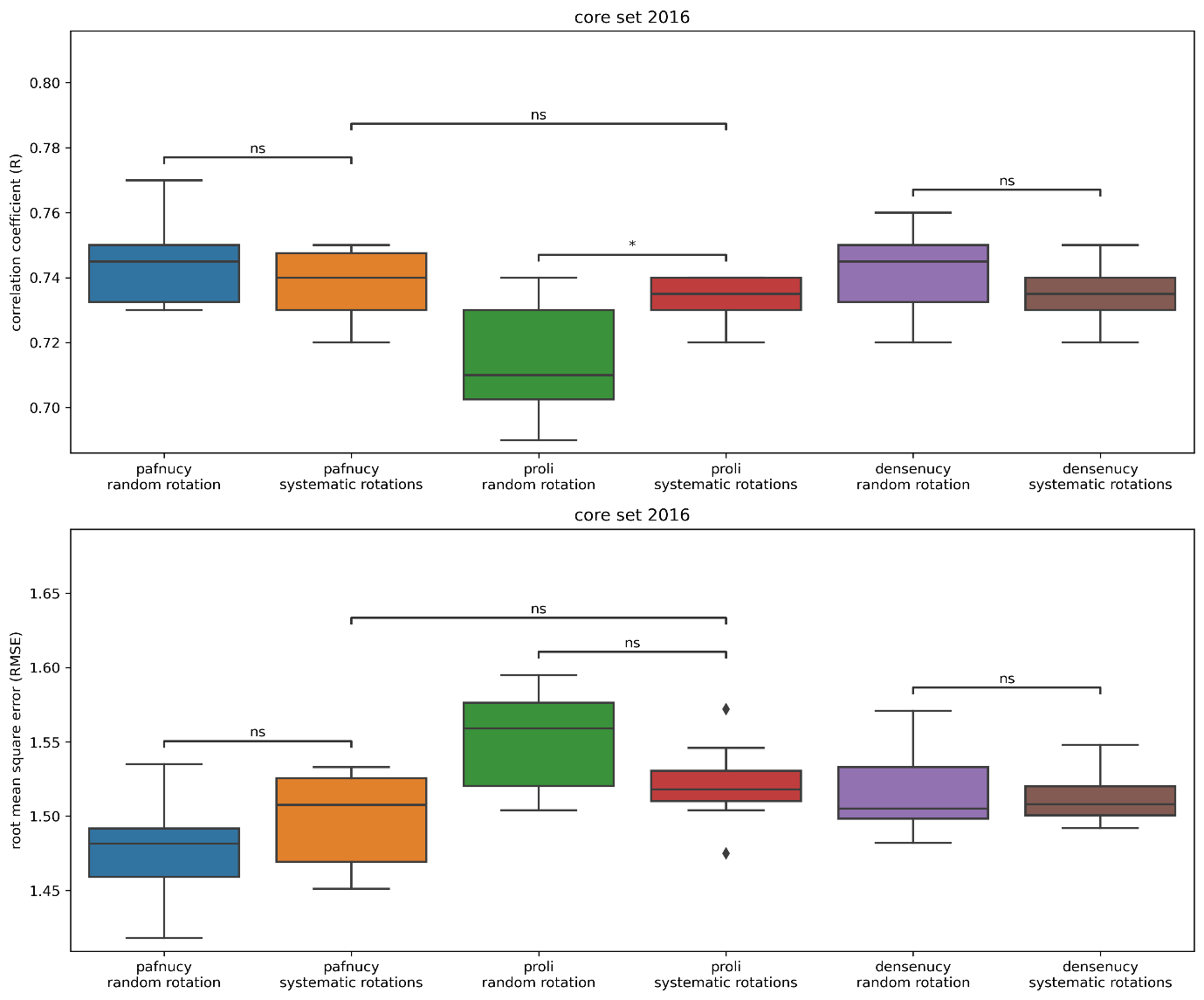


**Fig. S3. The performance of the models trained with Pafnucy, Proli and Densenucy, using random or systematic rotations.** Results are evaluated on the PDBbind v.2016 core set. For each training setting, the boxplot displays the results over 10 replicate models. The following p-values correspond to the annotations on the plots: ns: 5.00 × 10−2 < *p* ≤ 1.00 × 100, *: 1.00 × 10−2 < *p* ≤ 5.00 × 10−2, and ⧫ are possible outliers.


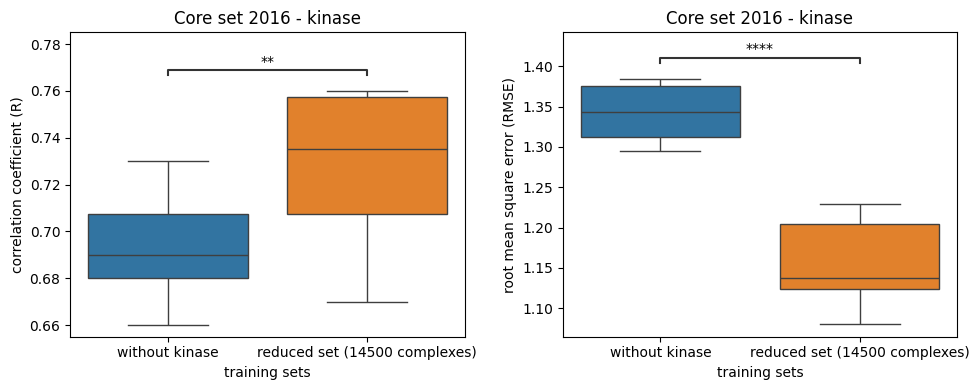


**B**

**A**


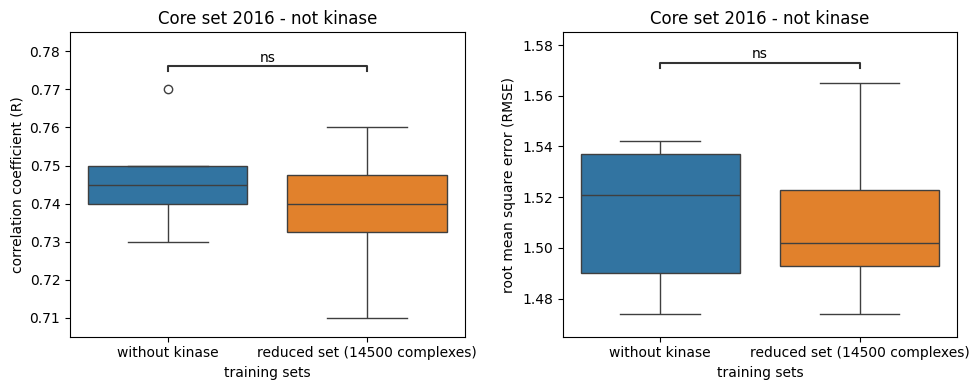


**Fig. S4. Evaluation of Pafnucy in a “Leave One Target Out” setting.** Models were trained on complexes excluding kinase family and on a subsampled set of the same size composed of randomly selected complexes (reduced set). A: models are evaluated on the complexes including kinase family from the PDBbind v.2016 core set. B: models are evaluated on the complexes without kinase family from the PDBbind v.2016 core set. The following p-values correspond to the annotations on the plots: ****: *p* ≤ 1.00 × 10−4, **: 1.00 × 10−3 < *p* ≤ 1.00 × 10−2, ns: 5.00 × 10−2 < *p* ≤ 1.00 × 100, and ○ are possible outliers. Models trained without kinases demonstrate significantly lower performance compared to other models when evaluated on the kinases from the PDBbind v.2016 core set. However, when evaluated on the remaining complexes in the PDBbind v.2016 core set, the performance of models trained without kinases is comparable to that of the other models.


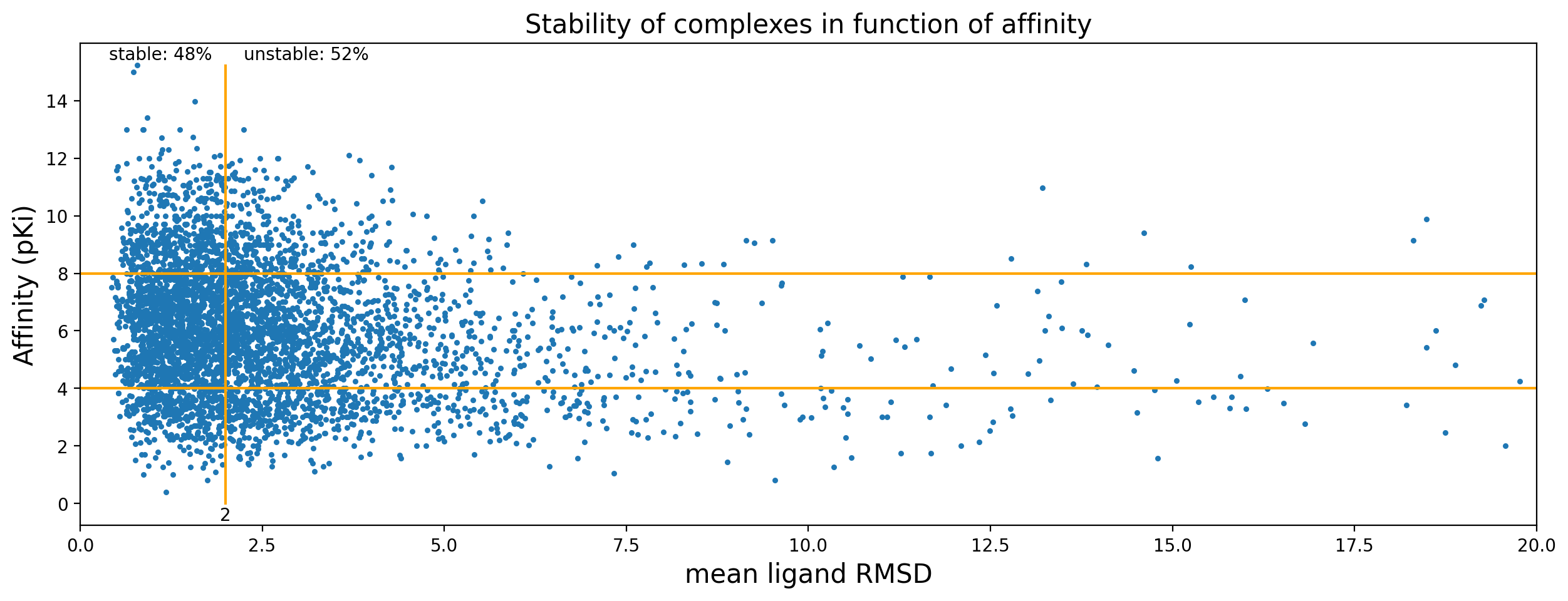


**Fig. S5. Relationships between affinity of protein-ligand complexes and the maximum RMSD per ligand during the simulation.** The analysis was conducted on the MDbind dataset. A stability threshold of 2 Å has been established. Complexes with an affinity lower than a pKi of 4 are classified as low affinity, while those with an affinity higher than a pKi of 8 are classified as high affinity.


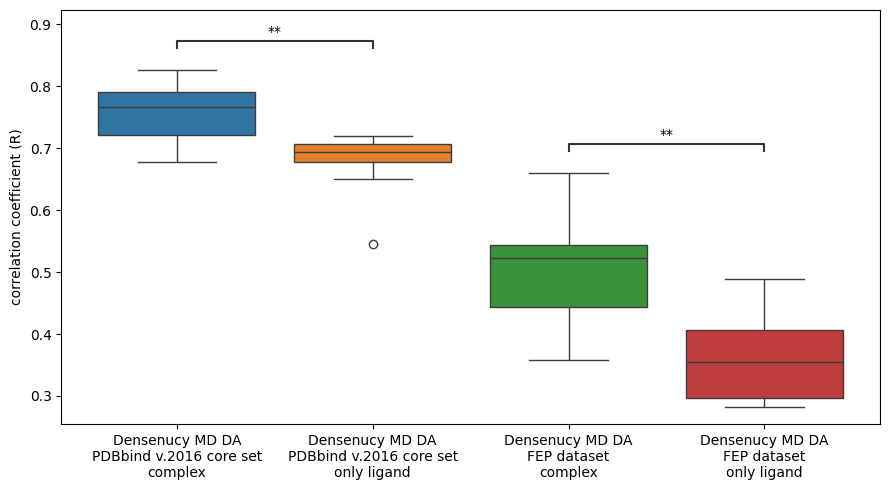


**Fig. S6. Comparison of Densenucy MDDA performance using complexes versus ligands on the PDBbind v.2016 core set and the FEP dataset.** The prediction gap on the PDBbind v.2016 core set has a median ΔRlig of 0.073, whereas it is 0.169 for the FEP dataset. The following p-values correspond to the annotations on the plot: **: 1.00 × 10−3 < *p* ≤ 1.00 × 10−2, and ○ are possible outliers.

Orengo CA, Michie AD, Jones S et al. CATH--a hierarchic classification of protein domain structures. Structure (London, England : 1993) 1997;5:1093-108.
